# A foundation model-based multi-instance learning framework for accurate prediction of lymph node metastasis in prostate cancer from whole slide images

**DOI:** 10.3389/fonc.2026.1775750

**Published:** 2026-03-04

**Authors:** Guang Zeng, Weiwei Li, Haonan Mei, Ran Du

**Affiliations:** 1 Division of Urology, The Central Hospital of Enshi Tujia and Miao Autonomous Prefecture, Enshi, Hubei, China; 2 Division of Nephrology, The Central Hospital of Enshi Tujia and Miao Autonomous Prefecture, Enshi, Hubei, China; 3 Hubei Selenium and Human Health Institute, The Central Hospital of Enshi Tujia and Miao Autonomous Prefecture, Enshi, China; 4 Hubei Provincial Key Lab of Selenium Resources and Bioapplications, Enshi, China; 5 The First Clinical College, Wuhan University, Wuhan, Hubei, China

**Keywords:** artificial intelligence, computational pathology, lymph node metastasis, multi-instance learning, N staging, prostate cancer

## Abstract

**Background:**

Nodal involvement (N stage) is a key prognostic factor in prostate cancer (PCa). Conventional imaging and histopathology often have limited sensitivity and inter-observer variability. AI-based computational pathology, using multi-instance learning (MIL) and foundation models, offers a promising approach for accurate and interpretable N stage prediction from H&E-stained whole slide images (WSIs).

**Methods:**

In this multicenter retrospective study, we developed a weakly supervised deep learning framework integrating MIL with domain-adapted foundation model encoders (UNI-v2, CONCH, ResNet-50) to predict N stage. WSIs from 280 RHWU patients were used for training and 306 TCGA patients for external validation. Attention heatmaps enabled interpretability, while transcriptomic analyses explored molecular correlates via differential expression and bioinformation analysis.

**Results:**

The UNI-v2-based model achieved the highest performance (AUC 0.879 in RHWU, 0.850 in TCGA), surpassing CONCH and ResNet-50. Attention heatmaps highlighted tumor-stromal interfaces and poorly differentiated tumor clusters. Transcriptomic analysis identified 94 differentially expressed genes; upregulated genes were enriched in cell cycle, and immune pathways, while downregulated genes involved ion transport and metabolism.

**Conclusions:**

This AI-MIL framework accurately predicts nodal involvement in PCa and provides biologically interpretable insights, supporting its potential as a precision oncology tool for risk stratification and treatment planning.

## 1 Introduction

Prostate cancer (PCa) is the second most frequently diagnosed malignancy and the fifth leading cause of cancer-related death among men worldwide (1). According to global cancer statistics, over 1.4 million new cases and 313,780 deaths were reported in 2025, with incidence continuing to rise due to population aging and improved diagnostic strategies (2). With an aging population worldwide, the volume of prostate biopsy specimens is expected to increase dramatically (3). Despite advances in imaging and systemic therapy, accurate staging remains essential for determining treatment strategies and predicting outcomes in PCa patients (4). Among the staging components, nodal involvement (N stage) is particularly critical, as lymph node metastasis is strongly associated with biochemical recurrence, distant progression, and reduced cancer-specific survival (5). Patients with positive lymph nodes often require multimodal treatment strategies, including androgen deprivation therapy, radiation, or systemic chemotherapy, in addition to radical prostatectomy (6).

Current evaluation of N stage primarily relies on preoperative imaging modalities such as computed tomography (CT), magnetic resonance imaging (MRI) (7), and prostate-specific membrane antigen positron emission tomography (PSMA-PET) (8). While PSMA-PET has demonstrated superior sensitivity compared with conventional imaging, its availability is limited in many regions, and its specificity remains imperfect. Histopathological examination of lymph nodes obtained during pelvic lymph node dissection (PLND) remains the gold standard; however, PLND is invasive and associated with perioperative risks. Furthermore, histological assessment of H&E-stained sections is subject to inter-observer variability, and small metastatic foci may be missed in routine practice (9). These limitations highlight the unmet need for accurate, scalable, and reproducible tools to predict nodal status in PCa.

Artificial intelligence (AI), particularly deep learning applied to digital pathology, has emerged as a transformative approach in oncological diagnostics (10). By leveraging whole slide images (WSIs), AI can capture subtle morphological patterns beyond the recognition of human observers. Multiple-Instance Learning (MIL), a weakly supervised paradigm, is particularly suitable for WSI analysis, as it requires only slide-level labels (e.g., N-positive vs. N-negative) without the need for exhaustive region-level annotation (11, 12). Recent developments in foundation models—large-scale pretrained encoders specifically adapted to histopathology—further enhance feature extraction, enabling generalizable and domain-specific representations of tissue morphology. Integrating MIL with pathology foundation models therefore represents a promising avenue for building robust, interpretable, and clinically deployable models for nodal staging in PCa. Beyond prediction, linking computational pathology findings with molecular alterations offers an opportunity to deepen biological understanding and identify novel biomarkers. Transcriptomic profiling from The Cancer Genome Atlas (TCGA) has revealed distinct molecular subtypes and immune landscapes of PCa, and integration with AI-derived predictions may provide mechanistic insights. In particular, exploring differentially expressed genes (DEGs), functional enrichment pathways, and immune infiltration profiles between N-positive and N-negative patients could illuminate tumor–immune interactions that drive metastatic dissemination (13).

In this study, we proposed a foundation model–based MIL framework for predicting N stage in prostate cancer from H&E-stained WSIs, using multicenter cohorts from RHWU and TCGA. Our objectives were threefold: (i) to develop and validate a weakly supervised model for slide-level prediction of nodal involvement, (ii) to provide interpretability through attention heatmaps highlighting predictive histological regions, and (iii) to perform exploratory bioinformatics analyses for uncovering potential molecular and immune correlates of nodal metastasis. This work aims to bridge the gap between computational pathology and translational oncology, ultimately supporting precision medicine in PCa management.

## 2 Methods

### 2.1 Patient cohorts

**Figure 1:**
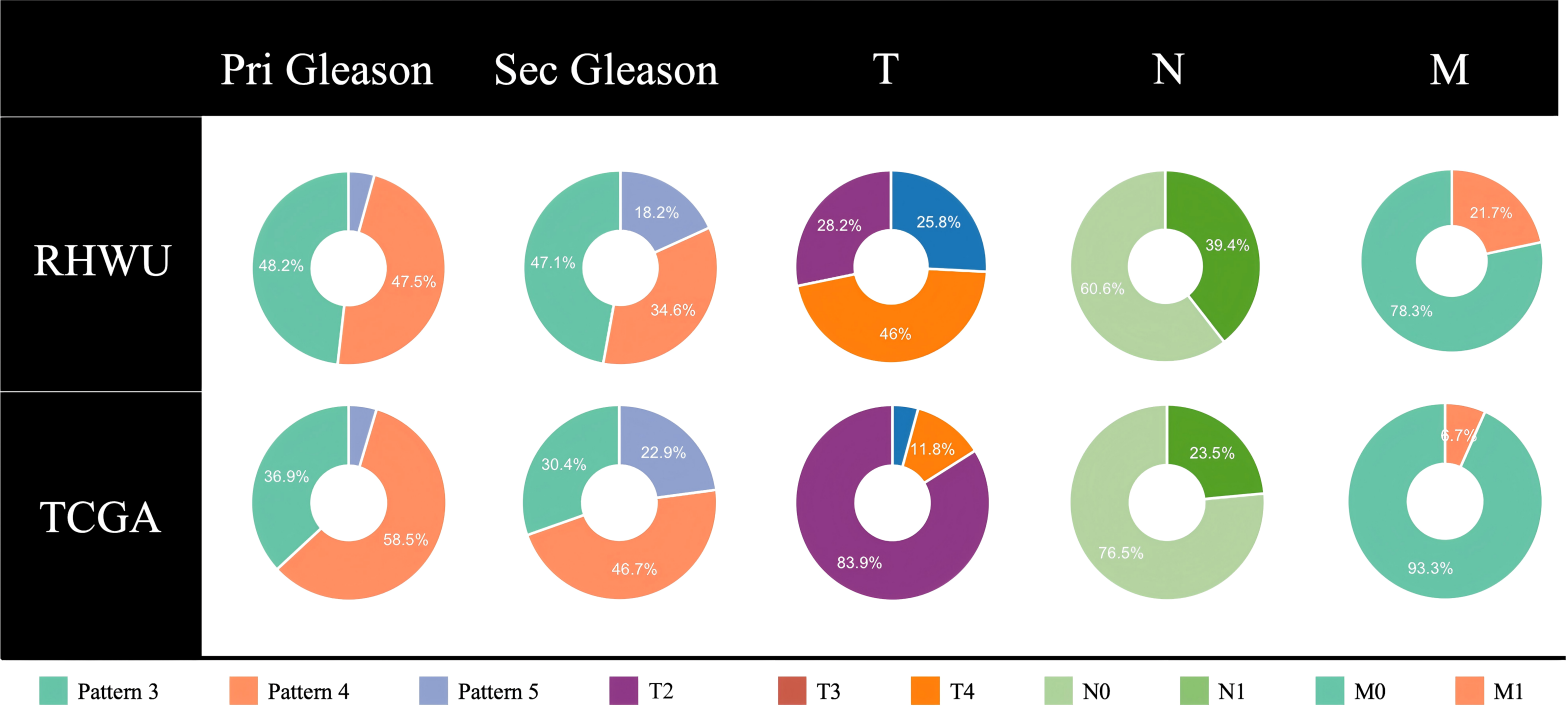
Demographic and staging systems in prostate cancer patients. Pri Gle for primary Gleason Score, Sec Gle for Secondary Gleason Score, T, N, and M for tumor TNM stage system.

This multicenter retrospective study analyzed two independent prostate cancer cohorts. The internal dataset comprised 280 patients who underwent radical prostatectomy at the Renmin Hospital of Wuhan University (RHWU, Wuhan, China) between 2018 and 2023. Eligible patients were required to have histologically confirmed prostate adenocarcinoma, complete clinicopathological information including postoperative nodal status, and diagnostic-grade hematoxylin and eosin (H&E)-stained WSIs containing more than 10% tumor area. Patients who had received neoadjuvant therapy prior to surgery were excluded. The external validation cohort consisted of 306 prostate adenocarcinoma patients from the TCGA-PRAD database, accessed in April 2024. This cohort provided both digitized WSIs and matched RNA sequencing data. Clinical information, including patient age, preoperative prostate-specific antigen (PSA) levels, Gleason grade, and TNM stage, was collected for all cases. Detailed cohort information is presented in **Figure 1** and **Table 1**.

**Table 1:** The distributions of demographic and clinicopathologic characteristics of patients.

	RHWU (N = 280)	TCGA (N = 306)
Age (Years)	74.1 ± 7.9	61.0 ± 6.8
Median pre-op PSA (ng/mL)		
tPSA	39.7 ± 52.8	na
cPSA	31.2 ± 18.6	na
TNM stage		
T2	28.2%	83.9%
T3	46.0%	11.8%
T4	25.8%	4.3%
N0	60.5%	76.4%
N1	39.4%	23.5%
M0	78.3%	93.3%
M1	21.7%	6.7%
Post-op GS (ISUP)		
2-6 (I)	27.0%	4.9%
7 (II)	20.5%	31.0%
7 (III)	16.4%	23.5%
8 (IV)	19.3%	16.0%
9-10 (V)	16.8%	24.5%

PSA, prostate-specific antigen; GS, Gleason score.

### 2.2 Overview of the study workflow

**Figure 2:**
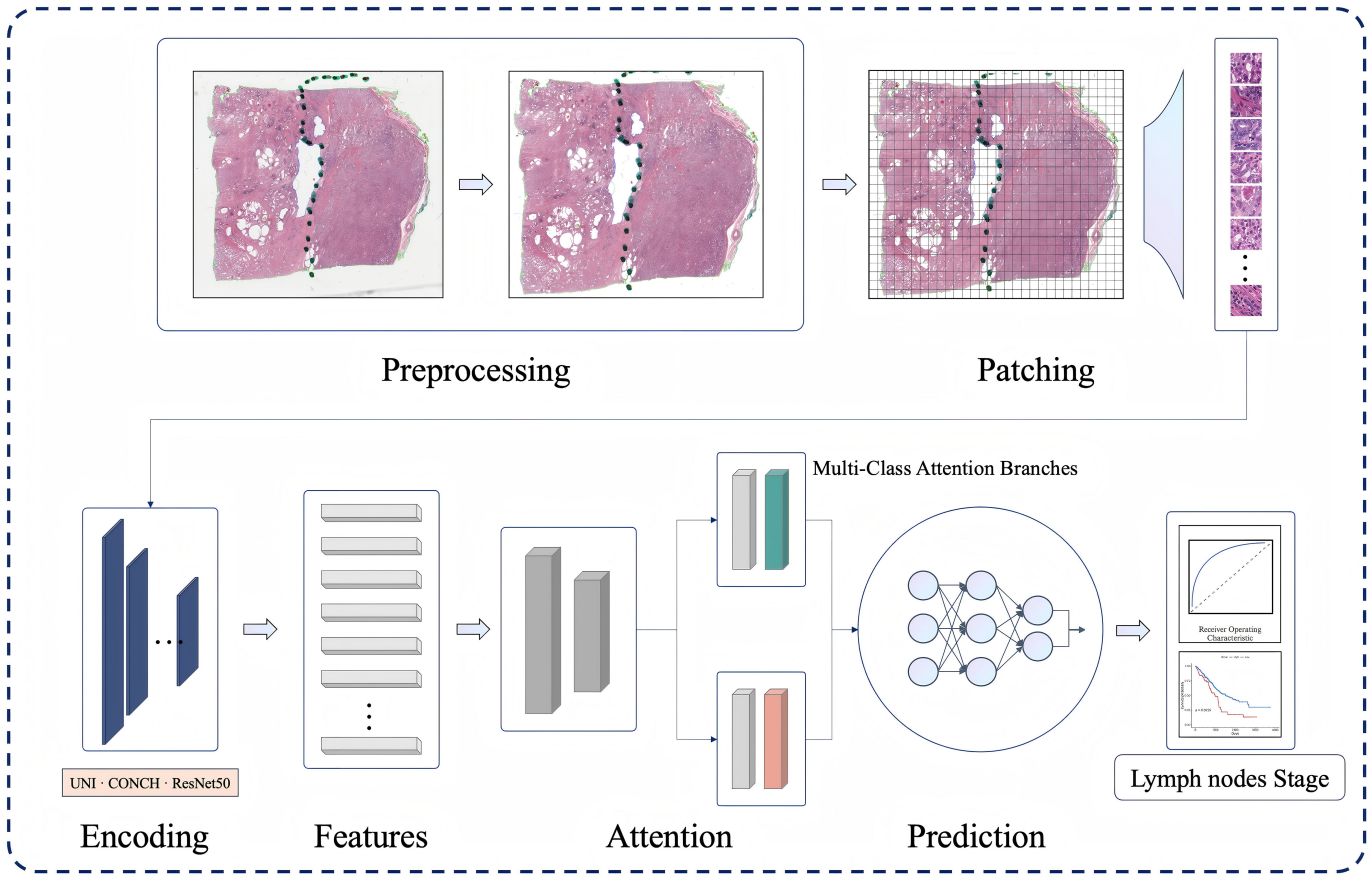
Flow chat of pretraining foundational model-based feature extraction and multi-instance learning for N stage predict in prostate cancer.

The workflow of the Prostate N stage prediction module is as follows and demonstrated in **Figure 2**. The training dataset comprises H&E-stained WSIs from the aforementioned cohorts, each labeled at the sample level according to pathological N stage (N0 or N1). Pathological images corresponding to each patient were collected from both cohorts. For the RHWU cohort, WSIs were digitized using a KF-PRO-020 digital slide scanner (KFBIO Co., Ltd.) at a magnification of 40× (0.25 μm per pixel). Each WSI was uniformly divided into 256 × 256 pixel patches, which were subsequently grouped into bags. These bags, together with their WSI-level N stage labels, served as input for training the MIL module. The trained model was then evaluated on both internal and external validation datasets, and its predicted N stages were compared against the pathologist-determined ground truth to assess performance and generalizability.

### 2.3 Preprocessing

For the RHWU cohort, H&E slides were digitized using the digital slide scanner (KFBIO Co., Ltd., Ningbo, China) at 40× magnification with a resolution of 0.12 μm/pixel. Images with quality issues such as air bubbles, uneven staining, severe fading, tissue folds, or out-of-focus areas were excluded from analysis. TCGA slides were obtained as pre-scanned digital images in SVS format. Each WSI was subdivided into non-overlapping patches of 256 × 256 pixels following stain normalization using the Macenko method in order to minimize color variation across samples. Patches containing more than 80% background area were discarded, which is the empirical criterion threshold that aims to balance noise reduction with preservation of informative tissue regions, and the remaining patches were aggregated into bags, which served as the analytical units for subsequent MIL.

### 2.4 Feature extract and deep learning methods

To extract meaningful representations from the image patches, three distinct pretrained encoders were selected based on their complementary strengths for histopathological analysis. Each model offers a different paradigm for visual representation learning: (i) UNI-v2, a histopathology-specific foundation model, leverages a self-supervised DINOv2 framework trained on large-scale histopathological datasets, which makes it particularly adept at capturing domain-relevant morphological patterns and robust to tissue-level variations (14). (ii) CONCH, a vision-language foundation model, is pretrained on paired histopathology images and biomedical text, enabling it to encode not only visual features but also semantically rich contextual knowledge highly relevant to diagnostic pathology (15). (iii) ResNet-50, a widely used convolutional architecture pretrained on ImageNet, was included as a well-established and general-purpose baseline to provide a performance benchmark and assess the added value of domain-specific pretraining. Each 256 × 256 patch was processed by the chosen encoder to yield a 1024- or 2048-dimensional feature vector, which were then aggregated at the whole-slide image (bag) level for downstream analysis (16).

The prediction of nodal involvement was performed using the Clustering-constrained Attention Multiple Instance Learning (CLAM) framework with a single-branch attention mechanism (clam-sb) (17). CLAM was selected over other MIL approaches (e.g., DSMIL, Transformer-based MIL) for several reasons. First, its gated attention mechanism is well-suited for histopathology tasks characterized by high intra-slide heterogeneity and weak supervision. Second, unlike Transformer-based MIL models which typically require larger datasets to avoid overfitting, CLAM has demonstrated robust performance on cohorts of moderate size comparable to ours. Third, CLAM explicitly models instance-level ambiguity by imposing clustering constraints, which helps identify both supportive and contradictory evidence within a slide, enhancing interpretability and robustness. Finally, CLAM provides direct visualization of attention scores, allowing transparent identification of the morphological regions most influential for the prediction, which aligns with our objective of model interpretability.

The RHWU cohort was used for model training and internal evaluation via five-fold cross-validation to preserve the class distribution in each fold during model development, with patients randomly assigned to training and test subsets in an 8:2 ratio. The TCGA-PRAD cohort served as an independent external validation set. Model performance was assessed at the patient level using multiple metrics, including accuracy, precision, recall, specificity, F1-score, and the area under the receiver operating characteristic curve (AUC), with 95% confidence intervals estimated by bootstrapping.

Feature extraction was performed using frozen pretrained encoders mentioned above. Encoder parameters were not fine-tuned during MIL training. Slide-level classification was optimized using a cross-entropy loss. The Adam optimizer was employed with an initial learning rate of 1 × 10^-4^; and a weight decay of 1 × 10^-5^. Training was conducted for a maximum of 20 epochs, and a dropout rate of 0.25 was applied to mitigate overfitting. Instance-level clustering was enabled without an explicit instance-level loss term. A total of eight representative instances (B = 8) were sampled from each bag during training, and the bag-level loss was weighted by a coefficient of 0.7. No class-weighted sampling strategy was applied at the patient level.

### 2.5 Visualization and interpretability

Model interpretability was examined through visualization of attention heatmaps. For a representative subset of cases (n=30 WSIs each from N0 and N1 groups, randomly selected), the tiles with the highest attention scores were extracted. These tiles, along with their corresponding low-attention control tiles from the same slide, were presented in a blinded and randomized order to two board-certified genitourinary pathologists. The pathologists were asked to qualitatively assess the presence of predefined histopathological features associated with aggressiveness (e.g., poorly differentiated clusters, nuclear atypia, stromal response) and to assign a subjective “suspicion for metastasis” score on a 3-point scale (low, intermediate, high).

To explore the potential molecular basis underlying the imaging-derived predictions, hypothesis-generating transcriptomic analyses were performed using RNA sequencing data from the TCGA-PRAD cohort. Patients were stratified according to pathological nodal status. Differential expression analysis was conducted using the edgeR package in R, with genes considered significant at an absolute log2 fold change greater than one and an adjusted p-value below 0.05. Identified differentially expressed genes (DEGs) were subjected to Gene Ontology (GO) and Kyoto Encyclopedia of Genes and Genomes (KEGG) enrichment analyses using the clusterProfiler package to elucidate the biological processes and pathways associated with nodal involvement. Genes strongly associated with nodal status were further evaluated for prognostic significance using Kaplan–Meier survival analyses, with overall survival (OS) as primary endpoint.

### 2.6 Statistical analysis and hardware

All statistical analyses were carried out using R software (version 3.5.1), Python 3.7, and SPSS 25.0 software (IBM Corp., Armonk, NY). The diagnostic performance of each model was assessed using confusion matrixes, receiver operating characteristic (ROC) curves, and the area under the curve (AUC). Descriptive analyses were presented using MEAN ± SD. A two-sided p-value < 0.05 was considered statistically significant. The training, testing, and inferring processes of the network were performed in PyTorch (Version 1.6, Python 3.8, Ubuntu 20.04.6 LTS, GeForce RTX4090).

### 2.7 Ethics approval and consent to participate

This study was conducted in accordance with the ethical standards of the Declaration of Helsinki and was approved by the RHWU Ethics Committee. Informed consent from the participants was waived by the institutional review board as the current study satisfied the following requirement for the waiver of informed consent: the research involved minimal risk to the participants.

## 3 Results

### 3.1 Model development and predictive performance

**Figure 3:**
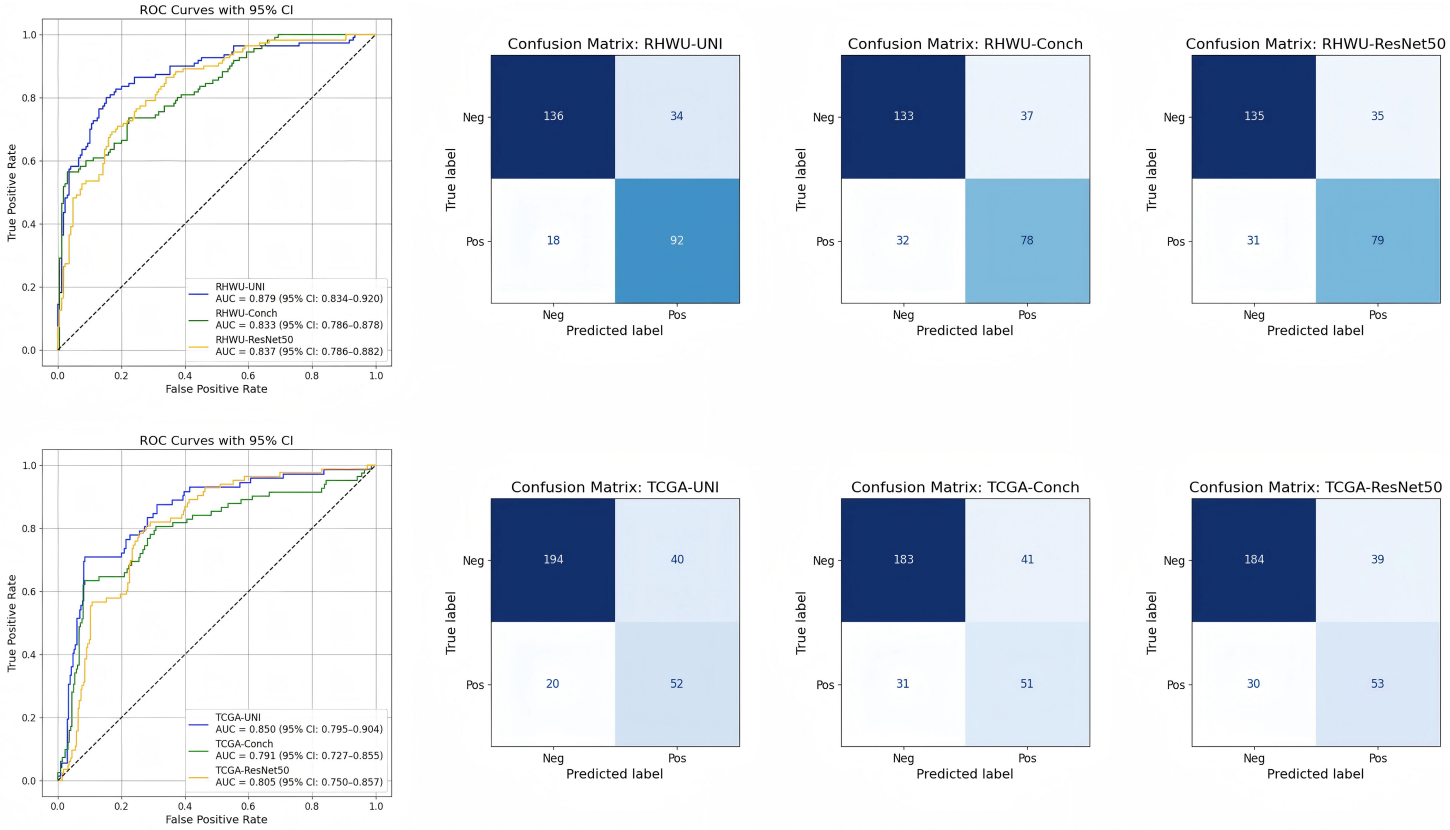
ROC curves and confusion matrices of different cohorts.

The proposed foundation model–based multi-instance learning framework successfully predicted nodal involvement in prostate cancer using H&E-stained whole slide images. Across five-fold cross-validation in the RHWU cohort, all encoders achieved robust classification performance, with the UNI-v2 encoder consistently outperforming CONCH and ResNet-50. Specifically, the UNI-v2 model achieved an average AUC of 0.879 (95% CI 0.834–0.920), accuracy of 81.4%, specificity of 73.0%, and F1 Score of 78.0%. By contrast, the CONCH-based model demonstrated an AUC of 0.833 (95% CI 0.786–0.878), while the ResNet-50 baseline reached 0.837 (95% CI 0.786–0.882). External validation on the TCGA cohort confirmed the generalizability of the models, with UNI-v2 again providing the strongest performance (AUC = 0.850, accuracy = 80.4%), followed by CONCH (AUC = 0.791) and ResNet-50 (AUC = 0.805). ROC and confusion matrix analysis illustrated consistent superiority of the UNI-v2 encoder across both datasets. The detailed results can be shown in **Table 2** and **Figure 3**.

**Table 2:** The efficacy of different cohorts in identifying N involvement in prostate cancer patients.

Cohort	Model	Acc	Pre	Recall	Spe	F1 Sco	AUC
RHWU	UNI-v2	0.814	0.730	0.836	0.800	0.780	0.879
	CONCH	0.754	0.678	0.709	0.782	0.693	0.833
	ResNet- 50	0.764	0.693	0.718	0.794	0.705	0.837
TCGA	UNI-v2	0.804	0.565	0.722	0.829	0.634	0.850
	CONCH	0.765	0.554	0.622	0.817	0.586	0.791
	ResNet- 50	0.775	0.576	0.639	0.825	0.606	0.805

Acc for accuracy, Pre for precision, Spe for Specificity, F1 Sco for F1 Score.

### 3.2 Interpretability analysis of attention mechanisms

**Figure 4:**
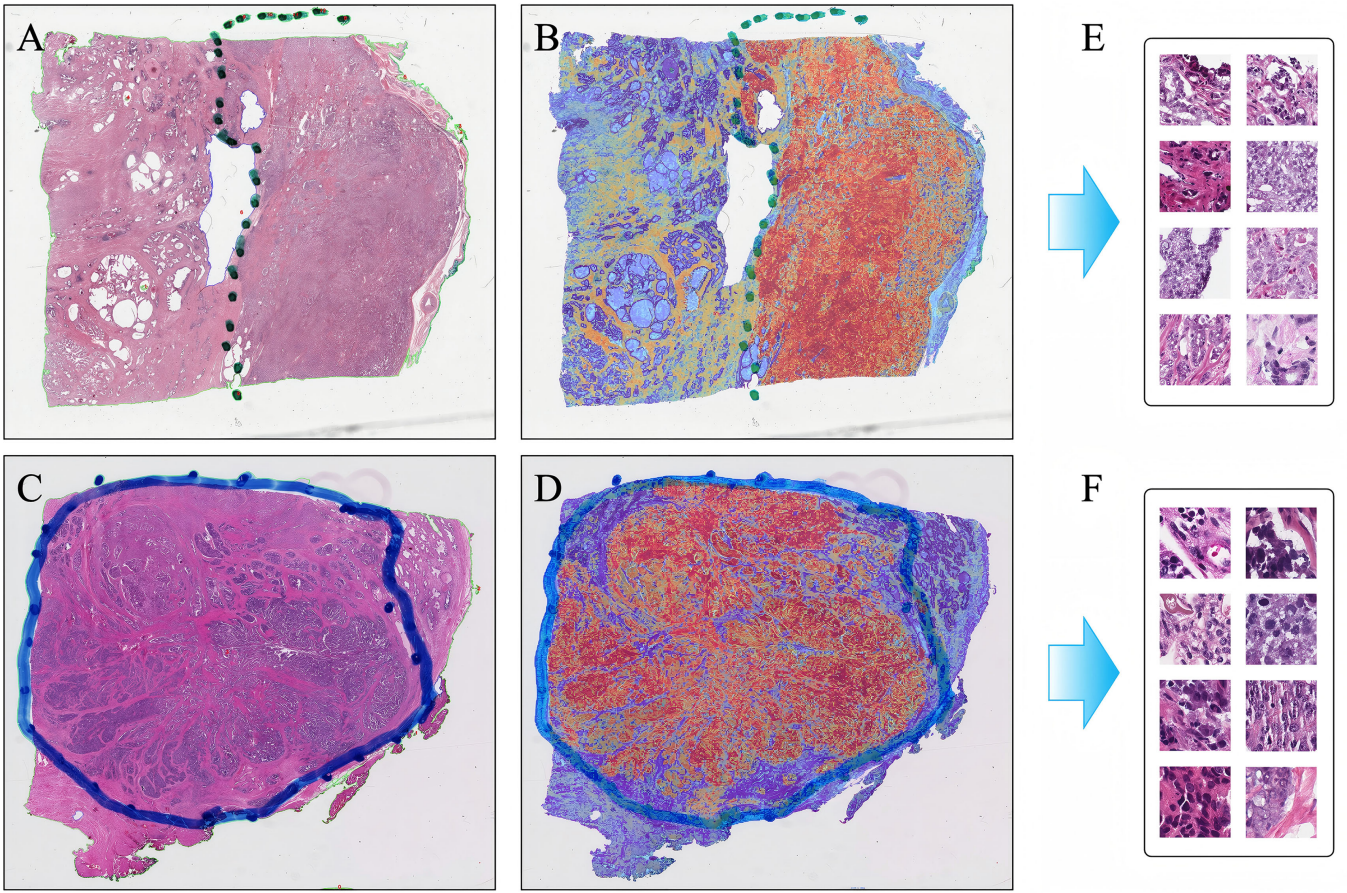
Representative whole slide images and attention heatmaps of N stage for prostate cancer patients. **(A, B)** N0 Patient, **(C, D)** N1 Patient **(E, F)** Patches of top 8 attention score.

To elucidate the histopathological features underlying model predictions, attention heatmaps were generated for each WSI (**Figure 4**). Based on the attention heatmaps, we retrospectively qualitatively analyzed the spatial distribution patterns of different cases. In patients with N-positive status, high-attention regions were concentrated at tumor–stroma interfaces, particularly in areas exhibiting poorly differentiated tumor clusters, nuclear pleomorphism, and mitotic activity. Stromal zones enriched with spindle-shaped fibroblasts and scattered lymphocytes were also frequently highlighted. Conversely, in N0 cases, the model focused on well-formed glandular structures with preserved polarity and minimal stromal reaction. Pathologist review of the top-ranked tiles confirmed the biological plausibility of the model’s focus, suggesting that the framework captured morphologic correlates of nodal metastasis. Representative WSIs with corresponding heatmaps demonstrated the ability of the model to localize regions of histological aggressiveness that may serve as surrogates for metastatic potential.

### 3.3 Differential gene expression and functional enrichment

**Figure 5:**
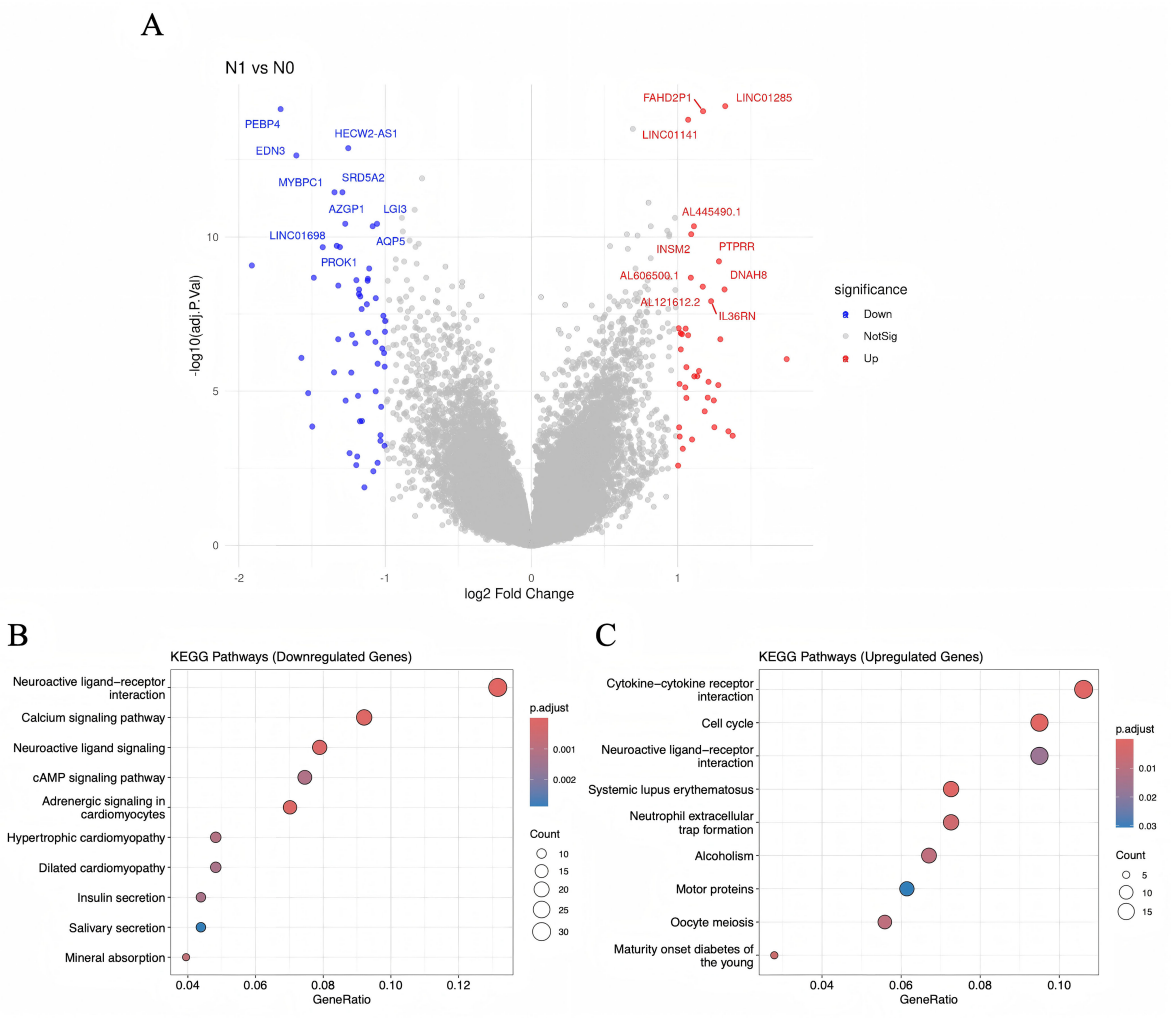
Volcano plot and KEGG enrichment analysis of DEGs.

To explore molecular correlates of nodal status, transcriptomic data from the TCGA cohort were analyzed in an exploratory, hypothesis-generating manner. A total of 94 genes were differentially expressed between N-positive and N-negative patients, based on thresholds of |log2 fold change| > 1 and adjusted p-value < 0.05. Volcano plot visualization demonstrated a distinct separation of upregulated and downregulated transcripts (**Figure 5**).

KEGG pathway analysis corroborated these findings (**Figure 5**). Upregulated pathways in the N1 group included Cell cycle, Systemic lupus erythematosus, Cytokine–cytokine receptor interaction, Neutrophil extracellular trap formation, and PI3K–Akt signaling, highlighting the interplay between cell proliferation, immune modulation, and metastatic potential. Conversely, downregulated pathways were enriched in Neuroactive ligand–receptor interaction, Calcium signaling, Adrenergic signaling in cardiomyocytes, and PPAR signaling, indicating suppression of neuroendocrine and metabolic functions in nodal disease.

**Figure 6:**
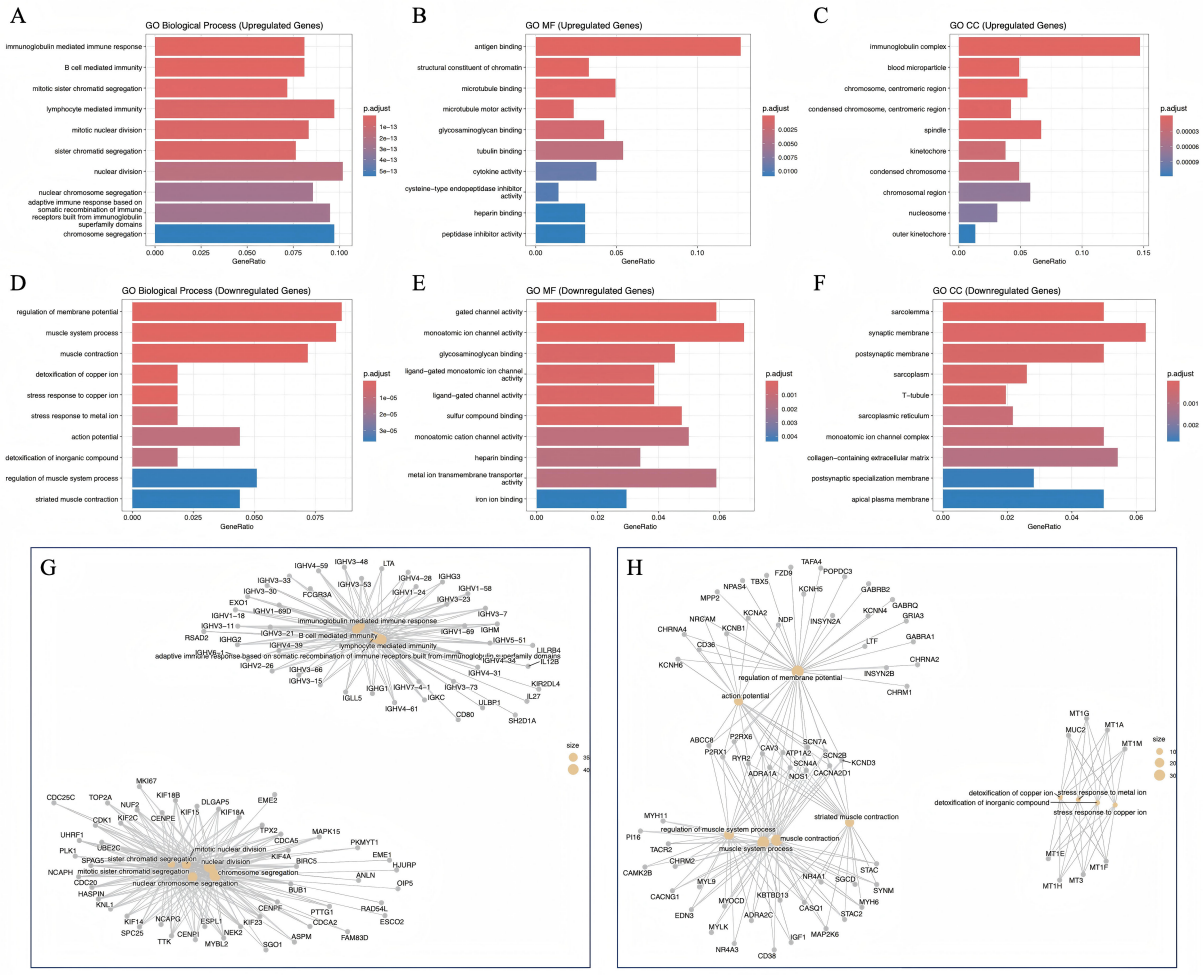
GO enrichment analysis and GO network of DEGs. **(A-C)** BP, MF, and CC of up regulated Genes; **(D-F)** BP, MF, and CC of dowm regulated Genes. **(G, H)** GO Enrichment Network of DEGs.

Gene Ontology (GO) enrichment analysis revealed distinct functional patterns among upregulated and downregulated genes across the categories of molecular function (MF), biological process (BP), and cellular component (CC), as shown in **Figure 6**. In terms of MF, the upregulated genes were mainly enriched in antigen binding, microtubule binding and motor activity, cytoskeletal motor activity, and peptidase inhibitor or regulator activity, indicating enhanced functions related to cytoskeletal dynamics and immune responses. Conversely, the downregulated genes were predominantly enriched in gated and voltage-gated ion channel activity, neurotransmitter receptor activity, and fatty acid or glycosaminoglycan binding, suggesting reduced transmembrane signaling and metabolic regulation.

Regarding BP, the upregulated genes were significantly associated with regulation of membrane potential, muscle system processes and contraction, response and detoxification to metal ions (copper, zinc, cadmium), heart contraction and blood pressure regulation, and extracellular matrix organization. In contrast, the downregulated genes were also enriched in pathways related to membrane potential regulation, ion transport, muscle contraction, and neuronal activity, implying functional alterations in ion homeostasis and muscular signaling under the studied condition.

For CC, upregulated genes were primarily located in the immunoglobulin complex, chromosomal and spindle-associated structures (including centromeric regions, kinetochores, and microtubules), reflecting enhanced mitotic and immune-related activities. Downregulated genes, however, were mainly distributed in the sarcolemma, synaptic and postsynaptic membranes, sarcoplasmic reticulum, and ion channel complexes, highlighting suppression of ion transport and neuromuscular transmission.

Collectively, these findings indicate that upregulated genes are mainly involved in immune activation, chromatin organization, and cytoskeletal remodeling, whereas downregulated genes are primarily associated with ion channel regulation, neuronal signaling, and metabolic processes, suggesting complementary functional shifts in response to the biological context examined. These analyses were exploratory in nature and aimed at generating hypotheses regarding the molecular landscape associated with AI-predicted and pathologically confirmed nodal status.

### 3.4 Prognostic relevance of nodal-associated genes

**Figure 7:**
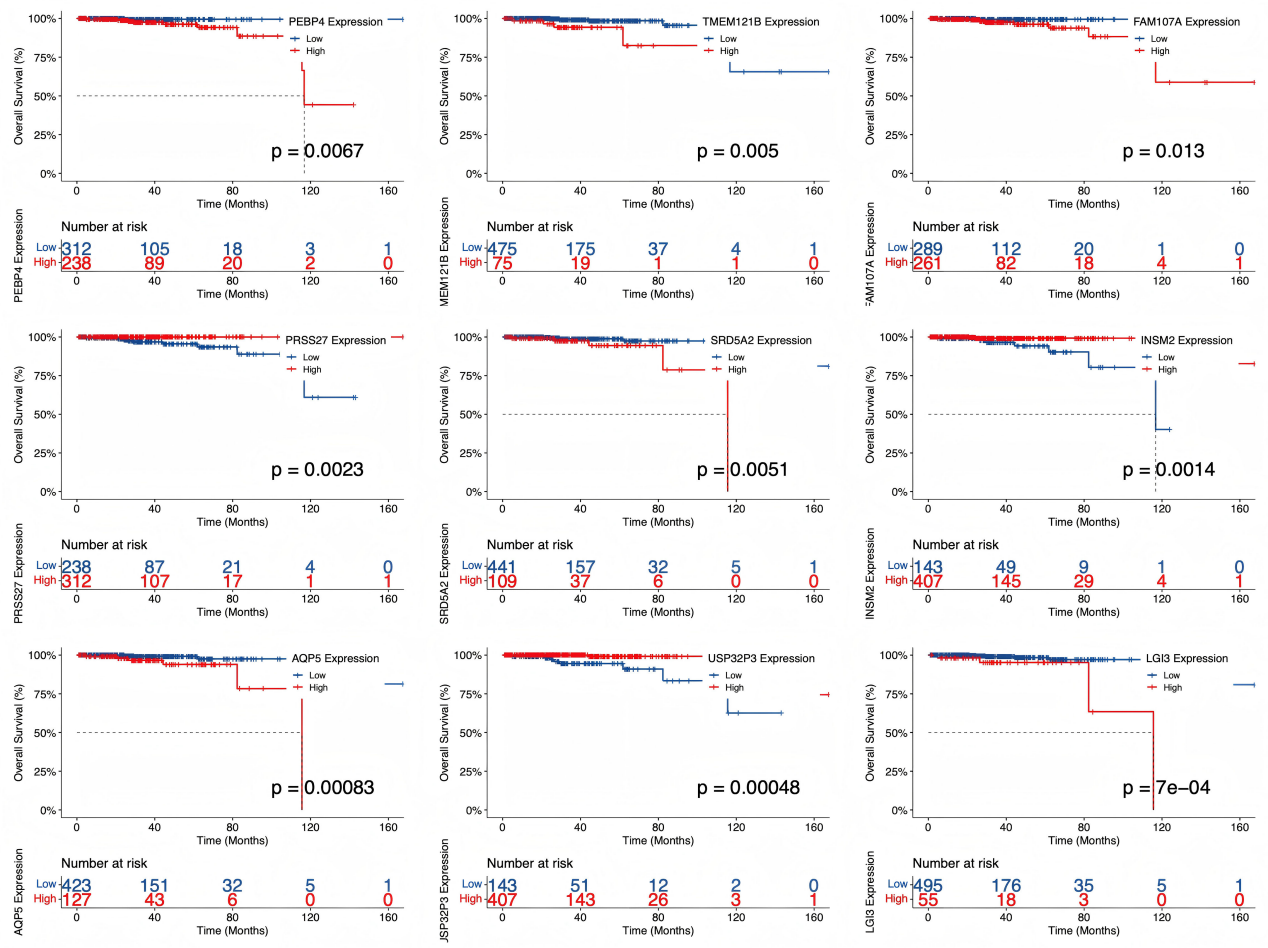
Kaplan–Meier survival curves of prostate N stage associated genes.

To assess the clinical impact of nodal-associated molecular alterations, Kaplan–Meier survival analyses were performed for selected DEGs with the strongest associations to nodal status (**Figure 7**). Several genes, including PEBP4, TMEM121B, FAM107A, PRSS27, SRD5A2, INSM2, AQP5, USP32P3, and LGI3 exhibited significant correlations with reduced overall survival (log-rank p < 0.01). These findings suggest a potential link between AI-identified morphological correlates of N stage and transcriptomic alterations associated with unfavorable prognosis. Further studies adjusting for clinical covariates are needed to validate the independent prognostic value of these genes.

## 4 Discussion

In this study, we developed and validated a pathology foundation model–based MIL framework for predicting nodal involvement in prostate cancer directly from hematoxylin and eosin (H&E)-stained whole slide images. Using a multicenter design incorporating both RHWU and TCGA cohorts, the proposed approach achieved high diagnostic accuracy, robust external generalizability, and meaningful biological interpretability. Beyond predictive performance, we further explored the molecular correlates of nodal status through integrative transcriptomic analyses, revealing differential gene expression patterns, functional enrichment in proliferative and invasive pathways, and immune infiltration profiles consistent with an immunosuppressive tumor microenvironment. Together, these findings underscore the potential of computational pathology to bridge histological morphology and tumor biology, offering clinically relevant insights into metastatic dissemination in prostate cancer.

Recent advances in computational pathology have demonstrated the potential of deep learning models to extract clinically relevant information directly from digitized histopathology slides. Deep learning models can automatically extract multi-level features from WSIs, enabling tissue region recognition, segmentation, and classification, thereby enhancing the automation and objectivity of prostate cancer pathology. Common CNN architectures such as U-Net, ResNet, VGG, and DenseNet have been widely applied to gland segmentation, tumor detection, and Gleason grading, achieving diagnostic consistency comparable to or exceeding that of pathologists (18–21). End-to-end deep learning approaches allow semantic segmentation and lesion classification directly from prostate histopathology images. U-Net and its variants perform well in gland and tumor segmentation, with Dice scores exceeding 0.90. Patch-based multi-layer CNNs can detect prostate cancer, assign Gleason grades, and assess perineural invasion, achieving AUC values up to 0.99. Moreover, weakly supervised and MIL methods effectively mitigate limited annotation issues, enabling high-performance tumor recognition using only slide-level labels (22–25). Self-supervised pre-trained foundation models for pathology, such as UNI, CONCH, and Virchow, have emerged as a research focus. Trained on massive cross-organ and cross-staining WSI datasets, these models possess strong feature extraction and generalization capabilities, achieving high downstream task performance with minimal annotated data. They support not only tumor detection and grading but also pathology report generation, image-text matching, and zero-shot retrieval, representing a systematic and platform-level advancement of AI in pathology.

MIL frameworks, including attention-based, CLAM-based approaches, DSMIL (26), and Transformer-based (27), have been successfully applied to predict key clinical endpoints such as lymph node metastasis, Gleason grading, and biochemical recurrence in prostate cancer, often achieving performance comparable to or exceeding that of human experts (25, 28, 29). We deliberately selected CLAM for the present study for several methodological and practical reasons. First, CLAM explicitly models instance-level heterogeneity within WSIs, which is particularly suitable for histopathology tasks characterized by weak supervision. Second, compared with Transformer-based MIL approaches, CLAM offers a more favorable trade-off between modeling capacity and data requirements. Transformer-based MIL models typically require substantially larger training cohorts to avoid overfitting, whereas CLAM has been shown to perform reliably on datasets of moderate size. Third, CLAM provides interpretability advantages through its attention scores and instance-level sampling strategy, allowing visualization of regions contributing most strongly to slide-level predictions.

Our results highlight several key contributions. First, the incorporation of domain-adapted foundation models, particularly the UNI-v2 encoder, substantially improved predictive performance compared to conventional ImageNet-pretrained backbones. This finding is consistent with emerging evidence that large-scale self-supervised learning on histopathology datasets yields representations that better capture the nuanced morphology of tumor tissue. Second, the use of CLAM-based MIL provided a weakly supervised yet interpretable framework for slide-level prediction. Unlike traditional convolutional neural networks, which often require extensive region-level annotations, our approach leveraged only slide-level labels while retaining spatial interpretability via attention heatmaps. These heatmaps consistently identified regions enriched in histological hallmarks of metastatic propensity, including nuclear atypia, poorly differentiated tumor clusters, and tumor–stromal interactions, which were corroborated by expert pathologists. Such interpretability is critical for clinical translation, as it allows pathologists to validate AI-driven predictions within established diagnostic paradigms.

The transcriptomic analyses, while exploratory, provided a valuable molecular context. Integrative bioinformatics analyses further contextualized the imaging-derived predictions. Differential expression analysis indicated that N-positive tumors exhibited transcriptomic alterations associated with metastatic potential. Specifically, several genes, including PEBP4, TMEM121B, FAM107A, PRSS27, SRD5A2, INSM2, AQP5, USP32P3, and LGI3, were significantly correlated with reduced overall survival (log-rank p < 0.01), highlighting their potential roles in disease progression, but the prognostic implications of the identified genes require validation in independent cohorts with multivariable models that account for standard clinical and pathological risk factors. These findings suggest that AI-identified morphological features of N stage are reflected at the molecular level. In addition, enrichment analyses revealed perturbations in pathways related to cell cycle regulation and extracellular matrix remodeling, consistent with mechanisms promoting tumor invasion and lymphatic dissemination.

In current clinical practice, lymph node involvement is primarily assessed through pelvic lymph node dissection (PLND) and imaging modalities such as PSMA-PET. When compared with existing clinical modalities for N staging, including MRI and PSMA-PET, our framework demonstrates several advantages. Imaging-based assessments, although non-invasive, often suffer from limited sensitivity, especially in detecting micrometastatic nodal disease. Histopathology remains the gold standard, yet manual review is labor-intensive and subject to inter-observer variability. By leveraging digitized WSIs and AI-based analysis, our approach provides a scalable and reproducible alternative that complements conventional diagnostics. Moreover, unlike imaging modalities that require specialized equipment and tracers, computational pathology can be implemented on widely available digital slides, facilitating broader clinical adoption. Despite the model’s high overall accuracy, false negatives could miss patients with lymph node involvement, while false positives might trigger unnecessary diagnostic procedures. Our model is best suited as a risk-stratification tool to identify low-risk patients, not as a definitive diagnostic, and that the operating threshold should be adjusted based on the clinical context—prioritizing sensitivity when ruling out metastasis is critical.

Despite its strengths, this study has limitations that warrant consideration. First, the RHWU cohort, although carefully curated, remains of modest size relative to the heterogeneity of prostate cancer. Larger, multicenter cohorts will be needed to further validate generalizability across populations and institutions. Second, while our attention-based interpretability offers valuable spatial insights, functional validation of highlighted regions was not performed, leaving the causal link between histological features and metastatic behavior to be further explored. Third, class imbalance is an inherent challenge in MIL tasks. Imbalance occurs at multiple levels, including the bag (patient/WSI) level and the instance patch level, where only a small fraction of patches within positive slides are truly discriminative, and the spatial level, where large areas of background or non-neoplastic tissue coexist with relatively sparse tumor or invasive regions. The TCGA cohort showed a less favorable class distribution, though the model was trained primarily on the RHWU cohort, which exhibited a relatively balanced class distribution and included a sufficient number of cases to support robust model training and feature learning, and its performance remained stable during external validation, suggesting that the learned representations were not overly driven by class frequency bias. Fourth, the RHWU training cohort has a higher proportion of locally advanced cases compared with the TCGA cohort. While this cohort heterogeneity may reflect real-world clinical practice, differences in case composition, stage distribution, and surgical strategies between cohorts could influence the generalizability of our model. Fifth, although transcriptomic analyses provided biological context, additional multi-omic integration (including genomics, epigenomics, and proteomics) could yield a more comprehensive understanding of metastatic progression. Finally, prospective evaluation in real-world diagnostic workflows will be essential to assess clinical utility, pathologist acceptance, and cost-effectiveness.

Looking ahead, several directions may enhance the translational potential of our framework. Expanding the sample size through multi-center data collection and incorporating explicit imbalance-aware strategies, such as class-weighted loss functions within the MIL classification objective. Incorporating multi-modal data, such as radiomics, clinical variables, and genomic alterations, could improve predictive accuracy and enable more personalized risk stratification. Expanding the scope to predict other clinically relevant endpoints, including biochemical recurrence, distant metastasis, and treatment response, may further increase clinical value. Moreover, the development of user-friendly interfaces that visualize AI-generated risk maps alongside pathology slides will facilitate integration into routine diagnostic practice and foster collaborative human–AI decision-making.

## 5 Conclusion

We present a robust and interpretable artificial intelligence framework that integrates pathology foundation models with multi-instance learning to predict nodal involvement in prostate cancer. Leveraging large-scale histopathology datasets from RHWU and TCGA, our approach demonstrated strong predictive performance, external generalizability, and meaningful biological interpretability. Transcriptomic and bio information analyses further revealed molecular mechanisms underlying nodal metastasis, highlighting potential biomarkers and therapeutic targets. These findings support the clinical potential of computational pathology as a decision-support tool for precision oncology, offering a scalable, reproducible, and biologically informed approach to prostate cancer staging and management.

## Data Availability

The original contributions presented in the study are included in the article/supplementary material. Further inquiries can be directed to the corresponding author.
